# Effect of Stir-Frying on Physicochemical and Functional Properties of Oat Protein Isolates

**DOI:** 10.3390/foods12142670

**Published:** 2023-07-11

**Authors:** Xia Wang, Yang Lei, Hamad Rafique, Liang Zou, Xinzhong Hu

**Affiliations:** 1 College of Food Engineering and Nutritional Science, Shaanxi Normal University, Xi’an 710119, China; wangxia5887@163.com (X.W.); leiyang19980105@163.com (Y.L.); hamad2020@snnu.edu.cn (H.R.); 2 School of Food and Biological Engineering, Chengdu University, Chengdu 610106, China; zouliang@cdu.edu.cn

**Keywords:** oat protein isolates, stir-frying, physicochemical properties, functionality

## Abstract

The heat treatment required for the deactivation of enzymes was carried out on crop species such as oats. Stir-frying, a frequently employed method for enzyme inactivation to preserve their desirable shelf life, can result in diminished nutritional value and protein degeneration. The mechanism by which stir-frying affects the oat protein remains largely unknown. Therefore, this study aimed to investigate the physicochemical and functional properties of the extracted oat protein isolates (OPI) at different stir-frying durations (0, 10, 20, and 30 min) at a temperature of 230 °C. The findings of this study demonstrated that stir-frying led to a decrease in the content of amino acids (AA), potentially attributed to the involvement of certain amino acids in the Maillard reaction. As the time of stir-frying increased, the secondary structure of OPI underwent changes: specifically, β-turns transformed into β-sheets. The process of protein denaturation and redistribution of chemical bonds resulted in an increase in the disulfide bond content of OPI, leading to aggregation, large particle size, and reduced digestibility. However, the water retention properties, foaming properties, and emulsification properties of OPI showed improvement. These findings provide valuable insights for the controlled and precise processing of oats and highlight the potential of OPI as a functional food.

## 1. Introduction

Oats (*Avena sativa*) are popular among all cereal crops due to their rich soluble dietary fiber and high amount (12–20%) of protein [1,2]. Oat protein is an excellent nutritional protein characterized by its hypoallergenic nature, high quality, and optimal amino acid composition. It possesses a high protein digestion-corrected amino acid score and contains substantial amounts of essential amino acids, particularly lysine and threonine, which play a crucial role in maintaining human health, regulating human physiological functions, and promoting bone development [3]. It is considered to be an effective way to obtain plant-based proteins [4]. Currently, oat protein is used in plant-based beverages and cosmetics, and it has a potential to play a significant role in the meat alternative market and the development of nutraceuticals as a new protein ingredient. However, the poor solubility of oat protein, due to its globulin content between 50% and 60%, affects other functional properties to some extent and limits its application. Oats contain various types of lipase, which can reduce their sensory storage quality [5]. Moreover, oat protein has a low molecular weight and lacks gluten proteins, resulting in a loose structure when mixed with water and difficulty in forming dough. Stir-frying is a commonly employed heat treatment method for oat-based foods, primarily aimed at modifying the thermal aggregation behavior of the protein to inactivate the enzymes, influence their functional properties, improve dough processing characteristics, and enhance the flavor of the product.

Nowadays, stir-frying is a key step in oat processing, which cannot only change the properties of oat kernels, but also affect the quality, structure, and functional characteristics of oat protein and its applications. It has been reported that heat treatment can lead to the formation of new enzyme-resistant cross-linking between proteins and carbohydrates or within protein molecules, resulting in impaired digestion, absorption, or utilization of amino acids, thus reducing the nutritional value of proteins [6]. Heat treatment affects protein solubility by affecting the content of free sulfhydryl and disulfide bonds. The content of free sulfhydryl and disulfide bonds undergoes a mutual conversion process. At temperatures below the protein denaturation threshold, the protein readily dissolves. However, when the temperature exceeds the denaturation threshold, the solubility of protein can be reduced due to the formation of disulfide bonds, facilitating polymerization [7]. Furthermore, heat treatment can affect the distribution of potential aggregate structures in the protein fractions. Zhao and Mine investigated the effect of heat treatment on oat globulin protein isolates [8]. Their results showed that short-term heating of the solution at 100 °C initially caused the globulin hexamers to break down into monomers, followed by aggregation of globulin into soluble aggregates due to protein denaturation. After heating at 100 °C for 60 min or longer, insoluble aggregates were formed, and induced aggregation occurred more rapidly at 110 °C. This aggregation behavior also causes changes in some functional properties of proteins. After thermal aggregation, the surface area of proteins and the availability of polar amino acids capable of binding water are weakened [9]. A previous study indicated that heat treatment can affect protein digestibility in various ways, changing the side chains of amino acids to reduce contact with certain digestive enzymes, forming transverse coupling, and reducing the digestibility of the whole protein molecule [10]. The stir-frying process, which is the core of oat processing and the traditional heat treatment method before milling, can affect the physicochemical and functional properties of OPI. At present, many studies have concentrated on the effects of the stir-frying process about the basic properties of oat kernels, whole oat flours, isolated starch, and the effect of different heat treatment methods such as baking and steaming on oat protein. Stir-frying is very critical to the nutritional quality and preservation of oat products. However, there is limited information on stir-frying time and how stir-frying affects the structure and function of oat protein. Therefore, it is necessary to understand how stir-frying affects the structure and function of oat protein.

In industrial production, stir-frying for 15–20 min (230 °C) is a common and essential processing technique, which makes it important to study the components’ changes during this procedure. In order to clarify the changes of protein structure and properties during stir-frying treatment, we conducted a comparative study: oats with different traditional stir-frying conditions (230 °C for 0, 10, 20, and 30 min) were selected to extract OPI. The objective of this study was to evaluate the effect of stir-frying on the physiochemical, structural, and functional properties of OPI and supply valuable insights for the controlled and precise processing of oats.

## 2. Materials and Methods

### 2.1. Materials

Fried oat grains provided by Inner Mongolia Yangufang, China, were used in this study. Prior to processing, the oats were subjected to moisture treatment to achieve a grain moisture content ranging from 20% to 24%. Subsequently, they were fried at a temperature of 230 °C for three different durations: 10 min, 20 min, and 30 min. At each time point, samples were collected and subsequently crushed using a laboratory universal grinder, including the bran component. Oat flour without stir-frying and including the bran component was used as a normal control in the study. All the reagents used in this study were of analytical grade.

### 2.2. Extraction of OPI from Oat Grain

#### 2.2.1. Milling and Defatting Process

The oat grains were ground by a laboratory mill (DFY-300C, Anting Scientific Instrument Factory, Shanghai, China) and underwent defatting following the methodology described in a previous study [11]. Briefly, oat flour was dispersed in Petroleum ether and allowed to stand at room temperature for 24 h. The dispersion was then centrifuged at 4000 r/min for 20 min. The supernatant was discarded and the solid pellet was allowed to dry overnight at room temperature.

#### 2.2.2. Extraction of OPI

The OPI were extracted from defatted oat flour according to a previously published method with slight modifications about the value of pH [12], which was more suitable for oat protein. The principle of extraction method is based on the high solubility of protein in alkaline solution and its properties of precipitation at isoelectric points. Briefly, 200 g of defatted flour was dissolved in distilled water at a ratio of 1:15 (*w*/*v*). After adjusting the pH to 10.0 with 1.0 M NaOH, the solution was stirred for 70 min at room temperature, followed by centrifugation (4000 r/min, 20 min) to remove any precipitation. The supernatant was collected and adjusted pH to 4.5 with 1.0 M HCl. After standing overnight, the protein was precipitated by centrifugation (4000 r/min, 15 min). The white precipitates were collected, redissolved in ultrapure water, and then the pH was adjusted to 7.0 with 1.0 M NaOH. All the extracted proteins were freeze-dried for 48 h to obtain OPI. The collected protein isolates were stored at 4 °C for further studies. Among them, stir-frying for 0 min, 10 min, 20 min, and 30 min, the extraction rates of OPI were 98.84%, 56.08%, 34.80%, and 28.11% and the corresponding purities were 92.74%, 89.68%, 82.01%, and 78.76%, respectively.

### 2.3. Structural Analysis of OPI

#### 2.3.1. Determination of Amino Acid Composition

The amino acid composition of OPI was analyzed by acid hydrolysis method (GB/T5009.124-2016) using an amino acid analyzer (HITACHIL-8900, Hitachi, Tokyo, Japan). Briefly, 100 mg of OPI powder was weighed and placed in the digestion tube, and 5 mL 6 M HCL (superior purity) was added. After 2 min of ultrasonication, the digestion tubes were purged with nitrogen and sealed. The sealed digestion tubes were placed in a microwave digestion instrument at 180 °C for 30 min, cooled, and then removed. After mixing, ultrapure water was added to a 50 mL flask. Then 1 mL of filtrate was taken and 1 mL of 0.5 M NaOH was added. After shaking well, the filtrate was added to a 10 mL flask with ultrapure water, and then filtered with a 0.45 μm inorganic filter membrane for instrumental determination. The flow rate of the pump was set at 0.1 mL/min, the temperature of the separation column was 57 °C, and the temperature of the reaction column was 135 °C. The analysis was performed by a fully automatic amino acid analyzer.

#### 2.3.2. Determination of Fourier Transform Infrared Spectroscopy (FTIR)

The secondary structure was characterized using FTIR spectroscopy (Tensor 27, Bruker, Germany). The FTIR of the oat protein isolate samples were measured according to an earlier report with a modification to sample mixing proportions [13]. The freeze-dried protein samples were mixed with dried spectral grade KBr (1:50, *w*/*w*), ground to powder in an agate mortar, and converted into thin slices. FTIR spectra were obtained in the wavenumber range of 400–4000 cm^−1^, with 32 scans and a resolution of 4 cm^−1^. Data were analyzed using Peakfit 4.12 (Systat Software, San Jose, CA, USA).

#### 2.3.3. Determination of the Molecular Weight Distribution

The weight-average molecular weight (M_w_), quantity-average molecular weight (M_n_), peak position molecular weight (M_p_), and polydispersity index (PDI) of the different oat protein isolate samples were determined by high-performance, size-exclusion chromatography (DAWN, Wyatt, SantaBarbara, CA, USA) combined with a multi-angle laser light scattering and refractive index detector (HPSEC-MALLS-RID) according to a previously reported method [8]. The OPI samples (2.0 mg/mL) were dissolved in the mobile phase (0.05 M PBS) and centrifuged (10,000 r/min, 10 min). The supernatant of the OPI samples was filtered by a 0.22 μm filter prior to analysis with the HPSEC system. The injection volume for each sample was 20 μL. The flow rate was 0.5 mL/min. The RI detector was calibrated over the linear concentration range with sodium chloride standards and operated at room temperature.

#### 2.3.4. Determination of Free Sulfhydryl (SH) and Disulfide Bond (SS) Content

The free SH and disulfide bond contents of OPI samples were determined according to the previously reported method with some modifications about sample dosage [14]. Briefly, a 0.5 mL (10 mg/mL) protein solution sample was prepared, and 2.5 mL (8 mol/L) urea solution and 0.02 mL DTNB (4 mg/mL) solution were added. The resulting mixture was incubated for 25 min at room temperature and then centrifuged for 10 min on a high-speed centrifuge (Shanghai, China). The absorbance value of the supernatant at 412 nm was measured by using a spectrophotometer; distilled water was used as a blank instead of samples. The SH content was calculated by

SH (μmol/g) = (73.53A_412_ × D)/C (1)

where A_412_ is the absorbance value measured at 412 nm; C is the concentration of the protein sample (mg/mL); D is the dilution factor and the value of D is 6.04.

A 0.2 mL (10 mg/mL) protein solution sample was prepared and 1.0 mL (10 mol/L) urea solution and 0.02 mL β-mercaptoethanol were added. The resulting mixture was reacted for 1 h at 25 °C and then 10 mL of 12% trichloroacetic acid (TCA) solution was added to react for 1 h. Then, after being centrifuged for 10 min at 4000 r/min, the precipitate was washed twice with 12% trichloroacetic acid (TCA) solution, dissolved in a 3.0 mL Tris-Gly-8 mol/L Urea solution, and then added with 0.04 mL DTNB solution. After the mixture was evenly mixed, it was incubated at 25 °C for 25 min, and then its absorbance value was measured at 412 nm. The SS content was calculated as the difference between total and free SH contents and was expressed as before in μmol/g of protein.

Total SH (μmol/g) = (73.53A_412_ × D)/C (2)

SS (μmol/g) = (Total sulfhydryl − SH)/2(3)

The value of D was 15.

#### 2.3.5. Determination of Average Particle Size

The OPI samples were dissolved in a 10 mM pH 7.0 sodium phosphate buffer, and the supernatant was diluted to a concentration of 0.2 mg/mL by magnetic stirring. It was centrifuged at 4000 r/min for 15 min, and then the supernatant was filtered by a 0.45 μm filter membrane, and the appropriate amount of filtrate was pipetted into the colorimetric dish for the laser particle size analyzer (S3500, Shanghai Xinrui Instrument Co., Ltd., Shanghai, China) [15].

### 2.4. Determination of Physicochemical and Functional Properties

The methods for measuring water holding capacity (WHC) [16], foaming capacity and stability [17], and emulsifying capacity and emulsion stability [18] were adapted from previously published methods.

The water holding capacity was determined by adding 80 mg OPI sample into 1.5 mL distilled water and stirring fully. The sample was oscillated every 5 min for 30 s. After 30 min, it was centrifuged at 8000 r/min for 10 min to remove free water and the water holding capacity was calculated by the following formula:

Water holding capacity (g/g) = (W_2_ − W_1_)/W_1_ (4)

W_1_ and W_2_ are the weights of the protein sample before and after water absorption, respectively.

The foaming capacity and stability were determined by adding 100 mL of 2% OPI solution into a 500 mL measuring cylinder and homogenizing for three times with a high-speed homogenizer at 10,000 r/min for 40 s; the foaming capacity and stability were calculated by

Foaming capacity (%) = (H_1_ − 100/100) × 100% (5)

Foaming stability (%) = (H_2_ − 100/H_1_ − 100) × 100% (6)

H_1_ and H_2_ are the heights of liquid level after homogenizing immediately and holding for 30 min, respectively.

The emulsifying capacity and emulsion stability were determined by preparing 50 mL 1% protein solution and adding 50 mL of soybean oil. Then, it was homogenized at 10,000 r/min for 2 min at room temperature and centrifuged at 1500 r/min for 5 min; the emulsifying capacity was calculated by

Emulsifying capacity (%) = (H_1_/H_2_) × 100% (7)

H_1_ and H_2_ are the heights of emulsification layer and total liquid, respectively. The emulsified sample was heated at 80 °C for 30 min, then cooled to room temperature and centrifuged at 1500 r/min for 5 min; the emulsion stability was calculated by

Emulsion stability (%) = (H_3_/H_1_) × 100% (8)

H_3_ is the height of emulsified layer after heating for 30 min.

### 2.5. Determination of Digestive Properties

The in vitro digestibility of oat protein was determined by a two-step pepsin digestion method [19], and the protein content of sample and precipitated protein content was determined by the Micro Kjeldahl nitrogen (HITACHIL-8900, Hitachi, Tokyo, Japan) method.

The specific operation was as follows: 1 g of the sample was weighed and dispersed in 0.1 M K_2_HPO_4_-KH_2_PO_4_ phosphate buffer to form a protein solution with a mass fraction of 5%. The solution was preheated in a water bath at 37 °C for 5 min, and the pH value was adjusted to 2.0, and 0.04 g of pepsin was added. The mixture was incubated on a constant temperature shaker at 37 °C for 1 h. Subsequently, the pH value was adjusted to 7.0 with 1 M NaOH, and 0.05 g of trypsin was added. The reaction was carried out on a constant temperature shaker at 37 °C for 2 h. Then, the reaction was terminated by a boiling water bath for 10 min. After centrifugation for 15 min, the precipitate was taken and freeze-dried in a refrigerator at 4 °C for backup.

In vitro digestibility = (crude protein content − precipitated protein content)/crude protein content × 100%(9)

### 2.6. Statistical Analysis

All analyses were performed at least three times. The data were expressed as means ± standard deviation and analyzed using SPSS 22.0 and Origin 9.0 software. The significant difference was set at *p* < 0.05 according to Duncan’s Multiple Range Test.

## 3. Results and Discussion

### 3.1. Structural Analysis of OPI

#### 3.1.1. Amino Acid Composition of OPI

The OPI before and after stir-frying (0, 10, 20, 30 min) all contained significant amounts of glutamate (Glu), Leucine (Leu), and aspartic acid (Asp), with the highest levels of glutamate (Glu) at 20.53%, 11.67%, 12.03%, and 12.00%, respectively. Cysteine (Cys) was hydrolyzed out, resulting in no detection due to being determined by the method of acid hydrolysis [20]. Among the eight essential amino acids, except for isoleucine (Ile) and histidine (His), the contents of other amino acids exceeded the respective percentages of the ideal protein recommended by FAO/WHO, indicating that the amino acid composition of OPI before and after stir-frying was relatively balanced with less nutritional loss. OPI plays an important role in maintaining the metabolic balance of the human body, consolidating and promoting immunity, and could be considered a protein with high nutritional value. Lys is the limiting amino acid of cereal, but its content is higher in oats, which has the effect of regulating human metabolic balance; Leu, as one of the most abundant functional amino acids in high-quality protein foods, can promote protein synthesis and improve energy metabolism in the body; Val plays an important role in promoting tissue growth and regulating blood sugar levels [21], both of which far exceed the recommended values, indicating that oat protein has excellent amino acid quality and high nutritional value.

Table 1 showed that the essential amino acid (EAA) content of OPI had no significant difference after processing, but the total amino acid (TAA) content decreased significantly with the increase in stir-frying time (*p* < 0.05). The TAA content of untreated protein was the highest at 77.19% and the lowest at 60.78% at 30 min of stir-frying, indicating that stir-frying would cause a certain loss of amino acids. This is consistent with previous findings [1]. On the one hand, this may be due to the fact that stir-frying for a long time has damaged amino acids. On the other hand, after stir-frying, the reduction in the amino acid content of the OPI may be due to stir-fry induced chemical modifications of OPI residues such as glycation, glycoxidation, and oxidation [22]. Some amino acids are involved in the Maillard reaction, among them being the decrease in lysine (Lys) content caused by the cross-linking of lysine side chains or the participation of ε-amino of lysine in the Maillard reaction. These results demonstrated that stir-frying had a significant influence on the amino acid composition of OPI and the amino acid content was lowest when stir-frying for 30 min.

#### 3.1.2. Secondary Structure Analysis of OPI

**Figure 1:**
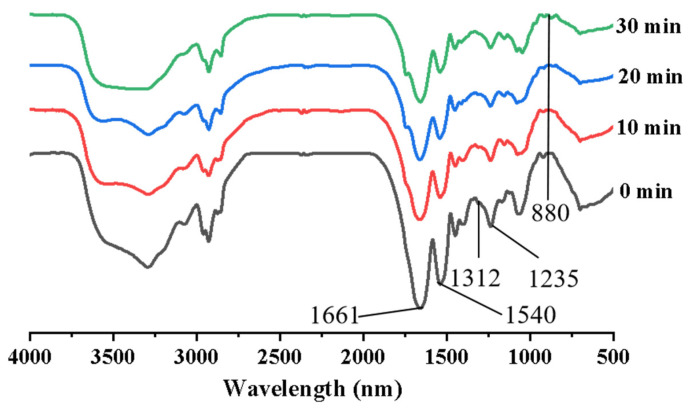
FTIR spectra of OPI at different stir-frying times.

Infrared spectroscopy can scan and identify characteristic functional groups in proteins and analyze the effect produced by stir-frying on protein structure. The results are shown in Figure 1. The structure of oat protein changed after stir-frying at different times. The absorption peaks of oat protein within the vibration region of amide 1 band (1700–1600 cm^−1^) were observed at 1661 cm^−1^, which was caused by the C=O stretching vibration. The absorption peaks within the vibration region (1600–1500 cm^−1^) of the amide Ⅱ band consistently appeared at 1540 cm^−1^, which were caused by the bending vibration of N-H and the C-N stretching vibration. Following the stir-frying treatment, the intensity of these absorption peaks decreased. Furthermore, in the amide Ⅲ band (1330–1220 cm^−1^), the absorption peaks were observed at 1312 cm^−1^ and 1235 cm^−1^, respectively, which were mainly caused by in-phase N-H bending vibration and C-N stretching vibration. The intensity of each characteristic absorption peak decreased significantly after the stir-frying process, potentially due to the consumption of specific functional groups in the Maillard reaction. In addition, as the duration of stir-frying increased, a new absorption peak emerged at 880 cm^−1^, representing the distinctive peak of polysaccharides. This observation suggests the presence of α-glycosides, and indicates the occurrence of the Maillard reaction during the stir-firing process, where the protein and polysaccharide became interlinked.

The amide band I region (1700–1600 cm^−1^) is the most sensitive range for analyzing protein secondary structure in infrared spectroscopy [23]. Therefore, the second derivatives of the amide I region were used to calculate the relative proportions of the secondary structures. The specific wavenumber ranges associated with various secondary structures are as follows: 1650–1660 cm^−1^ is α-helix, 1610–1640 cm^−1^ and 1690–1700 cm^−1^ are β-sheet, 1660–1690 cm^−1^ is β-turn, and 1640–1650 cm^−1^ is random coil [24]. It is worth noting that β-sheet and α-helix regions are typically located within the polypeptide chain, whereas β-turn arises from the folding of the polypeptide chain.

The main components of the secondary structure of oat protein are β-sheet and β-turn. The process of stir-frying can induce changes in the secondary structure of OPI shown in Table 2. With the increase of stir-frying time, the content of β-turn decreased, while the content of β-sheet, representing the ordered structure, increased, and the difference was significant (*p* < 0.05). Furthermore, extended processing time can lead to the formation of protein aggregates, transforming β-turns into β-sheets, and this transformation promotes the rise in β-sheet content, enhancing protein orderliness and stability. Usually, β-sheet represents the ordered structure of protein, which has a stronger hydrophobicity and a higher thermal denaturation temperature than α-helix; the more ordered the structure, the more stable the protein. After stir-frying for 30 min, the random coil structure disappeared and the β-sheet content accounted for the largest proportion (53.91%), indicating that the degree of protein aggregation was the largest and the order and stability were the highest at this time.

#### 3.1.3. Molecular Weights of OPI

Compared with the control sample, the M_w_, M_n_, and M_p_ of OPI increased with the increase in stir-frying time. The main component of OPI is globulin, which was found to dissociate into subunits and associate into high molecular weight aggregates [8]. This phenomenon can be attributed to the impact of high temperatures, which disrupt the natural protein during the stir-frying process. The protein was heated and extended, the hydrophobic residues in the protein molecules were exposed, and the hydrogen bonds between the protein molecules were broken and degraded into fragments with small molecular weights, which were repolymerized by disulfide bonds, so that the protein molecules were cross-linked to form larger aggregates. PDI indicates the polydispersity index. The lower the PDI, the more concentrated the distribution range of protein molecular weight. It can be seen from Table 3 that the PDI of OPI in the control group is the lowest and the molecular weight distribution is the most concentrated, while the molecular weight distribution of protein after stir-frying is relatively scattered.

#### 3.1.4. Content of Free Sulfhydryl and Disulfide Bond of OPI

**Figure 2:**
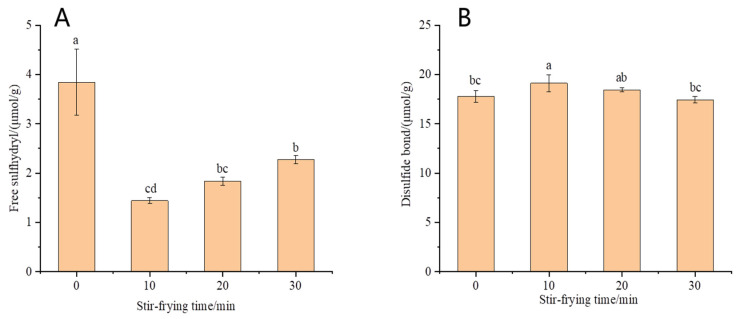
Contents of free sulfhydryl (**A**) and disulfide bonds (**B**) in OPI. Different letters indicate that the values are significantly different in the same test (*p* < 0.05).

The disulfide bond is a covalent linkage that exists in protein molecules and plays a crucial role in maintaining their structural integrity. Protein folding is essential for the formation of disulfide bonds, which undergo interconversion with a chemically active sulfhydryl group, thereby influencing the stability and functional properties of proteins. As can be seen from Figure 2, disulfide bond content of OPI first increased and then decreased as the stir-frying time increased, while the content of free sulfhydryl showed an opposite trend, although the difference was not statistically significant. The content of disulfide bonds and free sulfhydryl was the highest and the lowest, respectively, when stir-frying for 10 min, with significant differences. This may be attributed to the initial heating and unfolding of oat proteins during stir-frying, which exposed and facilitated the oxidation of free sulfhydryl groups, leading to the formation of intermolecular or intermolecular disulfide bonds, as well as cross-linking between protein molecules. Subsequent stir-frying resulted in protein molecule degradation into smaller particles, disulfide bond cleavage, and the re-exposure of internal free sulfhydryl groups, causing a decrease in disulfide bond content and an increase in free sulfhydryl content. In the process of stir-frying, the disulfide bond and sulfhydryl can be transformed into each other, which can stabilize the protein structure and improve its functional properties.

#### 3.1.5. Average Particle Size of OPI

**Figure 3:**
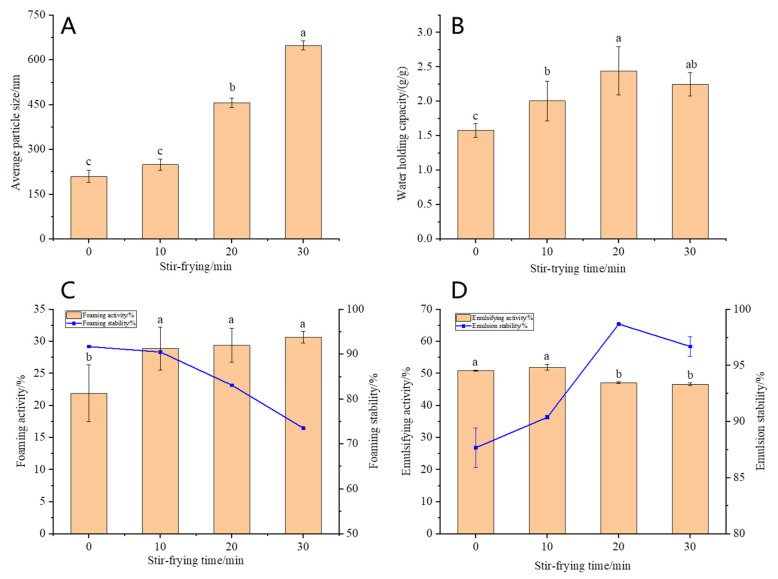
Average particle size (**A**), water holding capacity (**B**), foaming capacity and foaming stability (**C**), emulsifying capacity and emulsion stability (**D**) with different stir-frying times. Different letters indicate that the values are significantly different in the same test (*p* < 0.05).

The particle size distribution is one of the important indicators used to characterize the degree of protein aggregation. It can be observed from Figure 3A that compared with the control group, the average particle size of OPI increased from 209.63 nm to 648.63 nm after stir-frying and increased significantly with the increase in stir-frying time (*p* < 0.05), which corresponds to the trend of molecular weight. This indicates that stir-frying denatures and aggregates the OPI. The proteins formed a large number of thermal aggregates through intermolecular forces such as hydrogen bonds and disulfide bonds, and the aggregates were in the form of larger particles. This led to an increase in the average particle size of the protein.

### 3.2. Physicochemical and Functional Properties of OPI

The good water holding capacity of proteins can give foods good organoleptic and functional properties. The stir-frying treatment significantly improved the water holding capacity (Figure 3B) of OPI (*p* < 0.05). The reason was that stir-frying intensified the thermal motion of protein molecules, which resulted in a more uniform distribution of water in the protein and accelerated the absorption rate of water. In addition, OPI could be cross-linked to form large thermal aggregates with a higher stability after stir-frying, which can bind more water to fill the protein structure and significantly increase the water holding capacity. However, the water holding capacity of the oat protein isolate decreased slightly after stir-frying for 30 min, which may be due to the formation of some insoluble aggregates at this time, which affected the absorption rate and degree of polar groups in the protein into water. The water holding capacity of this study was similar to that of oat protein reported by Aleksei Kaleda [25] and slightly lower than that of soybean protein isolate [16].

As can be seen from Figure 3C, the foaming capacity of proteins increased after stir-frying, but there was no significant difference among the three stir-frying treatment groups. Stir-frying may increase the softness of protein peptide chains and make protein molecules adsorbed on the air–water interface, so that the network structure is easier to form. Stir-frying also exposes more hydrophobic amino acids, which in turn improves the ability of proteins to bind to air. Some studies have shown that the foaming capacity of protein may be related to the amount of hydrophobic amino acids free on the surface of the protein molecules [26]. After stir-frying, the OPI were heated and aggregated to form many aggregates of large molecular weight, which are sufficient to cover the surface of bubbles and form a rigid membrane structure around them to cause the generation of more bubbles. After stir-frying for 10 min, the foam stability of OPI did not change significantly but decreased with the increase in stir-frying time. When stir-frying for 30 min, the foam stability decreased by 20%.

The emulsifying capacity and emulsion stability of proteins are related to the flexibility and hydrophobicity of molecules. Good flexibility helps molecules rearrange at the interface. At the initial stage of stir-frying, the molecular flexibility of OPI increased, resulting in a slight increase in emulsifying capacity. With the further process, protein denaturation and thermal aggregation formed many large aggregates, which led to a decrease in molecular flexibility and solubility, and only a few protein molecules could diffuse to the oil–water interface in a short period of time, resulting in a significant decrease in emulsifying capacity (*p* < 0.05). After stir-frying, emulsion stability increased (Figure 3D). It has been found the emulsion stability of OPI could be increased and the emulsification activity decreased by moderate heating [27]. This is consistent with the results of this study. The difference was significant when stir-frying for 20 min (*p* < 0.05).

### 3.3. Digestive Properties of OPI

**Figure 4:**
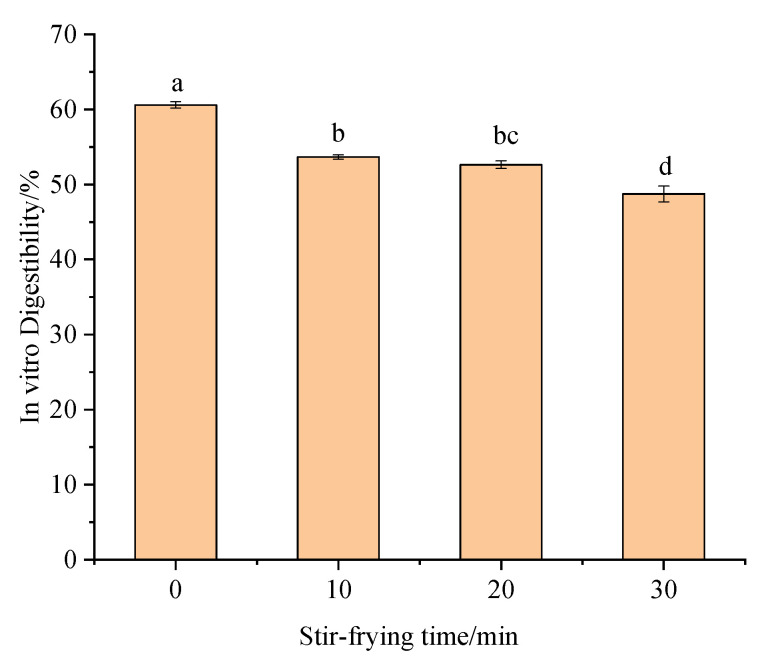
In vitro gastrointestinal digestion of oat protein isolates. Different letters indicate that the values are significantly different in the same test (*p* < 0.05).

Protein digestibility is an important indicator of protein nutritional value and is influenced by the protein source, ingredient particle size, maturity, protein modification, and processing methods [28]. After stir-frying, proteins changed their structural and functional properties shown in Figure 4, leading to changes in digestive properties. Compared with the control samples, the in vitro digestibility of OPI after stir-frying (0, 10, 20, 30 min) was significantly decreased, and the unheated oat protein was easier to digest and absorb [29]. On the other hand, heating causes the oat protein to break apart. This exposed the hydrophobic group, making the hydrophobic effect stronger and making the protein less soluble, all of which affect digestion. In addition, disulfide bonds are an important factor affecting in vitro digestibility. When oat protein is heated at a high temperature, a large number of disulfide bonds are formed between protein chains, and cross-linking between disulfide bonds leads to the formation of large molecular weight and more stable aggregates that block the enzyme’s action and reduce the digestibility [30]. The protein β-sheet content increased after stir-frying, and a few studies showed that the β-sheet content was negatively correlated with in vitro digestibility, which was consistent with the experimental results. In order to make better use of the high-quality protein in oats, mild heat treatment can be used to process oats.

## 4. Conclusions

The results suggested that stir-frying treatment of oats had substantial effects on oat protein isolates’ characteristics. Stir-frying did not change the content of essential amino acids (EAA) in oat protein isolates, while the total amino acid (TAA) content decreased significantly. Stir-frying treatment also impacted the protein’s secondary structure by transforming β-turn to β-sheet, which made the protein order and stability increase. Such unique structural properties of oat protein after processing have better water holding capacity, higher foaming ability, and emulsion properties, which can promote the processing characteristics of oat dough to a certain extent. Moreover, protein molecules were cross-linked to form larger aggregates, M_w_ of oat protein isolates was increased, and digestibility of oat protein isolates decreased after stir-frying. In conclusion, our findings suggested that oat protein isolates with better functionality could be prepared with proper stir-frying time. The selection of processing time is vital to improving the value and promoting the utilization of oats in the food industry. Stir-frying also has important effects on other components, i.e., β-glucan and starch, so it is particularly important to study the changes in these components and their interactions in the future. Analyzing the changes in nutrient content during the stir-frying process can provide valuable insights into the health benefits and potential drawbacks of consuming stir-fried oats.

## Figures and Tables

**Table 1:** Amino acid composition of OPI.

Amino Acid		Content (%)				
		0 min	10 min	20 min	30 min	FAO/WHO Standard (Adult)
EAA	Isoleccine (Ile)	0.32 ± 0.05 ^b^	0.32 ± 0.02 ^b^	0.32 ± 0.02 ^b^	0.56 ± 0.03 ^a^	1.3
	Leucine (Leu)	5.42 ± 0.09 ^a^	5.31 ± 0.06 ^a^	5.26 ± 0.04 ^a^	5.26 ± 0.20 ^a^	1.9
	Valine (Val)	3.89 ± 0.77 ^a^	4.50 ± 0.03 ^a^	4.41 ± 0.02 ^a^	3.60 ± 0.09 ^a^	1.3
	Lysine (Lys)	2.31 ± 0.10 ^a^	1.93 ± 0.10 ^ab^	1.82 ± 0.18 ^ab^	1.67 ± 0.29 ^b^	1.6
	Phenyiaianine (Phe)	3.05 ± 0.67 ^a^	3.17 ± 0.32 ^a^	3.00 ± 0.18 ^a^	3.30 ± 0.14 ^a^	1.9
	Methionine (Met)	2.57 ± 1.85 ^a^	3.14 ± 0.09 ^a^	3.04 ± 0.07 ^a^	1.14 ± 0.01 ^a^	1.7
	Threonine (Thr)	1.21 ± 0.02 ^b^	1.08 ± 0.01 ^c^	1.10 ± 0.02 ^c^	1.49 ± 0.02 ^a^	0.9
	Histidine (His)	1.76 ± 0.14 ^a^	1.33 ± 0.20 ^a^	1.36 ± 0.13 ^a^	1.29 ± 0.33 ^a^	1.6
NEAA	Asparagine (Asp)	4.97 ± 0.16 ^a^	4.507 ± 0.04 ^a^	4.27 ± 0.03 ^a^	5.08 ± 0.60 ^a^	-
	Serine (Ser)	2.71 ± 0.05 ^a^	2.62 ± 0.04 ^a^	2.43 ± 0.01 ^a^	2.74 ± 0.43 ^a^	-
	Glutamic acid (Glu)	20.53 ± 0.34 ^a^	11.67 ± 0.11 ^b^	12.03 ± 0.15 ^b^	12.00 ± 0.63 ^b^	-
	Giycine (Gly)	3.32 ± 0.09 ^a^	3.22 ± 0.03 ^ab^	3.02 ± 0.05 ^ab^	2.75 ± 0.37 ^b^	-
	Alanine (Ala)	10.56 ± 0.47 ^a^	10.06 ± 0.09 ^ab^	9.62 ± 0.01 ^b^	9.82 ± 0.38 ^ab^	-
	Tryptophan (Tyr)	3.50 ± 0.41 ^a^	4.01 ± 0.07 ^a^	3.56 ± 0.03 ^a^	3.46 ± 0.20 ^a^	-
	Arginine (Arg)	3.94 ± 0.21 ^a^	3.14 ± 0.38 ^a^	3.31 ± 0.13 ^a^	3.40 ± 0.45 ^a^	-
	Proline (Pro)	7.15 ± 2.53 ^a^	5.23 ± 0.12 ^ab^	5.26 ± 0.01 ^ab^	3.23 ± 0.01 ^b^	-
EAA		20.53 ± 2.02 ^a^	20.68 ± 0.60 ^a^	20.43 ± 0.32 ^a^	18.30 ± 0.71 ^a^	-
TAA		77.19 ± 0.12 ^a^	65.13 ± 0.30 ^b^	63.93 ± 0.70 ^b^	60.78 ± 0.96 ^c^	-

EAA, essential amino acid; NEAA, non-essential amino acid; TAA, total amino acid. Note: Mean ± standard deviations. Different letters indicate that the values are significantly different in the same test (*p* < 0.05).

**Table 2:** Secondary structure compositions of OPI at different stir-frying times.

Stir-Frying Time/min	Secondary Structure (%)			
	α-Helix	β-Sheet	β-Turn	Random Coil
0	17.56 ± 0.02 ^b^	17.12 ± 1.82 ^c^	53.00 ± 2.56 ^a^	12.32 ± 4.39 ^a^
10	15.64 ± 3.16 ^b^	31.00 ± 6.69 ^b^	41.06 ± 3.73 ^b^	12.30 ± 0.20 ^a^
20	16.06 ± 1.58 ^b^	31.26 ± 3.15 ^b^	37.78 ± 1.99 ^b^	14.90 ± 2.74 ^a^
30	22.88 ± 0.81 ^a^	53.91 ± 0.05 ^a^	23.21 ± 0.75 ^c^	/

Note: Mean ± standard deviations. Different letters indicate that the values are significantly different in the same test (*p* < 0.05).

**Table 3:** Molecular weights distribution of OPI at different stir-frying times.

Stir-Frying Time/min	M_w_ (g/mol)	M_n_ (g/mol)	M_p_ (g/mol)	PDI
0	(4.285 ± 1.217%) × 10^5^	(4.205 ± 1.269%) × 10^5^	(4.336 ± 1.079%) × 10^5^	1.019 ± 1.758%
10	(4.840 ± 2.024%) × 10^5^	(4.742 ± 2.087%) × 10^5^	(4.675 ± 1.846%) × 10^5^	1.021 ± 2.907%
20	(6.725 ± 1.040%) × 10^5^	(6.459 ± 1.055%) × 10^5^	(6.587 ± 1.000%) × 10^5^	1.041 ± 1.481%
30	(9.350 ± 1.269%) × 10^5^	(8.825 ± 1.266%) × 10^5^	(8.977 ± 1.280%) × 10^5^	1.059 ± 1.792%

## Data Availability

The datasets generated for this study are available on request to the corresponding author.
